# Fleas and lesions in armadillo osteoderms

**DOI:** 10.1111/joa.13842

**Published:** 2023-03-02

**Authors:** Alan Boyde, David Mills, Agustin Manuel Abba, María Cecilia Ezquiaga

**Affiliations:** ^1^ Dental Physical Sciences, Barts' and The London School of Medicine and Dentistry Queen Mary University of London London UK; ^2^ Centro de Estudios Parasitológicos y de Vectores (CEPAVE) UNLP‐CONICET La Plata Argentina

**Keywords:** bone resorption, carapace, *Chaetophractus*, *Dasypus*, insect trace fossils, *Tolypeutes*, *Tunga perforans*, Xenarthra

## Abstract

Armadillos are bitten by several species of flea. Females of the genus *Tunga* penetrate the epidermis and when in place are fertilised by males, after which the abdomen swells enormously to form a ‘neosome’. Within the *penetrans* group, *T. perforans*, makes lesions that perforate the osteoderms within the integument to form ~3 mm diameter cavities occupied by a discoid neosome. We examined these lesions in carapace material from animals which had died in the wild to see whether we could recruit evidence as to how they may be generated, either by the insect or by the host. We studied one species without such lesions, the nine‐banded armadillo *Dasypus novemcinctus*, and two species with, the greater hairy armadillo *Chaetophractus villosus* and the southern three‐banded armadillo *Tolypeutes matacus*, both showing the characteristic ‘flea bite’ holes in the external surfaces of the osteoderms. Samples were studied by three‐dimensional backscattered electron mode scanning electron microscopy and X‐ray microtomography. Both methods showed resorption pit complexes in the external surfaces of the osteoderms characteristic of those made by osteoclasts in active bone resorption. Lesions involved both the syndesmoses (sutures) between adjacent bones and the central regions of the osteoderms. Many lesions showed extensive repair by infilling with new bone. We conclude that the *T. perforans* neosome creates a local host response which causes bone resorption, creating the space in which it can grow.

## Graphical abstract

Study of lesions caused by flea's ‘bites’ in the bones on the backs of armadillos show that they are actually made by the host's own osteoclasts, not by the flea's own masticatory apparatus. Panels (a, c, e) are reconstructions from high‐contrast resolution X‐Ray Microtomography and (b, d, f) backscattered electron mode scanning electron microscopy views with matched orientation.

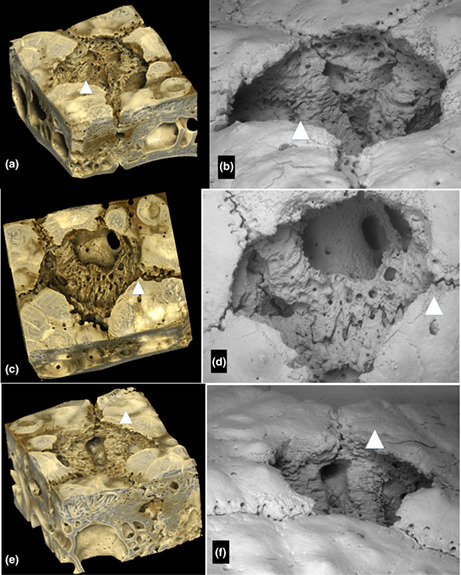

## 1 INTRODUCTION

The xenarthrans (Mammalia, Xenarthra), with only 38 living species, is the least diversified of the four major groups of placental mammals and the only group which originated in South America (Feijó et al., 2022; Gibb et al., 2016; Meredith et al., 2011). Amongst extant xenarthrans, only armadillos have dermal ossifications (=osteoderms). Practically all xenarthran living species are restricted to the Neotropics and only the nine‐banded armadillo (*Dasypus novemcinctus*) inhabits the southern part of the United States (McDonough & Loughry, 2018).

The armadillos are grouped into two families, Chlamyphoridae and Dasypodidae (Gibb et al., 2016) and both have an osseous carapace covering the dorsum, head and—in most species—the tail: they have large anterior claws for digging and are omnivores (McDonough & Loughry, 2018; Superina & Abba, 2018).

Armadillo osteoderms have extrinsic (Sharpey) fibre bone at their junctional (suture facing) peripheries and on the outside, and are secondarily remodelled to osteonal and trabecular bone internally. The whole arrangement of the separate osteoderms is held together with Sharpey fibres running from one bone to the next (Alves et al., 2017; Krmpotic et al., 2009, 2015; Scarano et al., 2019; Sire et al., 2009; Vickaryous & Hall, 2006).

Armadillos are bitten by several species of flea, most of which belong to the families Malacopsyllidae and Tungidae (Ezquiaga & Lareschi, 2012; Ezquiaga et al., 2021; Sanchez et al., 2020). Both groups are semi‐penetrating or directly penetrating, in which females attach to their host with serrated, sharp maxillary laciniae, showing great adaptability to their host (Hopkins & Rothschild, 1953; Smit, 1987).

Females of the genus *Tunga* penetrate the epidermis and when in place are fertilised by males, after which the abdomen increases in linear size by up to 10 times to form a ‘neosome’ (Marshall, 1981; Pampiglione et al., 2009).

The genus *Tunga* includes 13 species of flea distributed around the tropics (De Avelar et al., 2012). Smit (1962) divided the genus into two groups, the ‘penetrans group’ and the ‘caecata group’. Within the penetrans group, a new species, *T. perforans* (Ezquiaga et al., 2015), causes lesions which perforate the bones within the integument of its host. However, lesions identical to those attributed at *T. perforans* have been documented in at least six armadillos species: *Chaetophractus vellerosus*, *C. villosus*, *Tolypeutes matacus*, *Zaedyus pichiy*, *Euphractus sexcinctus* and *Priodontes maximus* (Ezquiaga et al., 2015, 2020). The neosome of this flea is discoid and compressed anteroposteriorly. Males move freely on the host's body (Ezquiaga et al., 2015).

The aim of this work was to investigate the lesions found on the osteoderms of armadillos parasitised by *T. perforans*. We hypothesised that the cavities eaten into the bone might be generated by the recruitment of the host's osteoclasts and that they would resemble Howship's lacunae, being formed of multiples of small resorption pits.

## 2 MATERIALS AND METHODS

We studied osteoderm samples from two armadillo species which showed the characteristic ~3 mm diameter lesions in the external surfaces of the osteoderms: namely, one *C. villosus* (greater hairy armadillo, the most common armadillo in the pampas grassland, Abba & Vizcaíno, 2011) and two samples of *T. matacus* (southern three‐banded armadillo, a species capable of rolling into a complete ball in self‐defence, Superina & Abba, 2018). These were adult specimens found dead (by natural reasons or subsistence hunting) and of unknown sex. The specimens examined are published in Ezquiaga et al. (2020). We did not kill any animal for this study. The permission number of the field study issued by Dirección de Fauna y Áreas Naturales Protegidas de la Provincia del Chaco, Argentina was ‘disposition 12/2017’. There were many lesions in each of the samples. We also studied bones from one road kill sample of *Dasypus novemcinctus*, the nine‐banded armadillo, which has no such lesions.

We used three‐dimensional (3D) backscattered electron mode scanning electron microscopy (BSE–SEM; Boyde, 2019), for which samples were studied after treatment with sodium hypochlorite bleach to remove residual adherent soft tissue and contaminant soil or dust particles, washed, dried and imaged uncoated at 20 kV, 50 Pa chamber pressure, using a Zeiss EVO‐MA10 SEM (Zeiss: all the SEM figures except Figure S11a).

For X‐ray microtomography (XMT) we used the QMUL MuCat2 (Davis et al., 2012) system, at 90 kV, 180 μA, with beam hardening filters of 1.2 mm Al and 0.05 mm Cu, ~2500 projections and 6 s exposures. Samples consisting of many adherent osteoderms were strengthened with Araldite epoxy resin and cut to smaller pieces to isolate regions containing the bony lesions and to allow imaging at 10 μm voxel resolution. Whilst scanning, the samples were supported on perspex rods with adhesive wax (Wax 6969; Poth Hille & Co). Reconstruction and calibration of the grey levels of the reconstructed data were performed as detailed by Davis et al. (2012). Volumetric rendering used Drishti scientific visualisation software (Limaye, 2012) to explore the volumetric data, produce 3D images and also to guide the cutting and trimming of the samples for further 3D BSE–SEM imaging. BSE–SEM and XMT Drishti reconstructions were correlated. After XMT, in some cases, bone was dissolved from Araldite to leave a negative cast of the lesion for SEM study. Other normal bone tissue was included in polymethylmethacrylate (PMMA), and the bone then dissolved to make 3D casts of internal space structure (Boyde, 2019).

For the *D. novemcinctus* material, some tissue was additionally embedded in PMMA, and blocks cut and polished to produce flat sectioned surfaces which were carbon coated and used for both qualitative and quantitative compositional contrast BSE‐SEM (Boyde, 2019) using a Zeiss DSM962 SEM (Zeiss; Figure S11a); anorganic samples giving 3D topographic contrast were also carbon coated and imaged with the DSM962 (Figure S11b); and ground sections were prepared for multiple rotation polarised light microscopy (Kirby et al., 2020; Figure S11c).

## 3 RESULTS

With both 3D BSE–SEM and XMT and in both *Chaetophractus* and *Tolypeutes*, many stages of formation and repair of ‘flea bite’ holes were observed, from extensive osteoclastic resorption of both Sharpey fibre bone and internal remodelled osteonal and trabecular bone to new bone formation and filling of cavities (Figures 1, 2, 3; Figures S5–S10).

**FIGURE 1:**
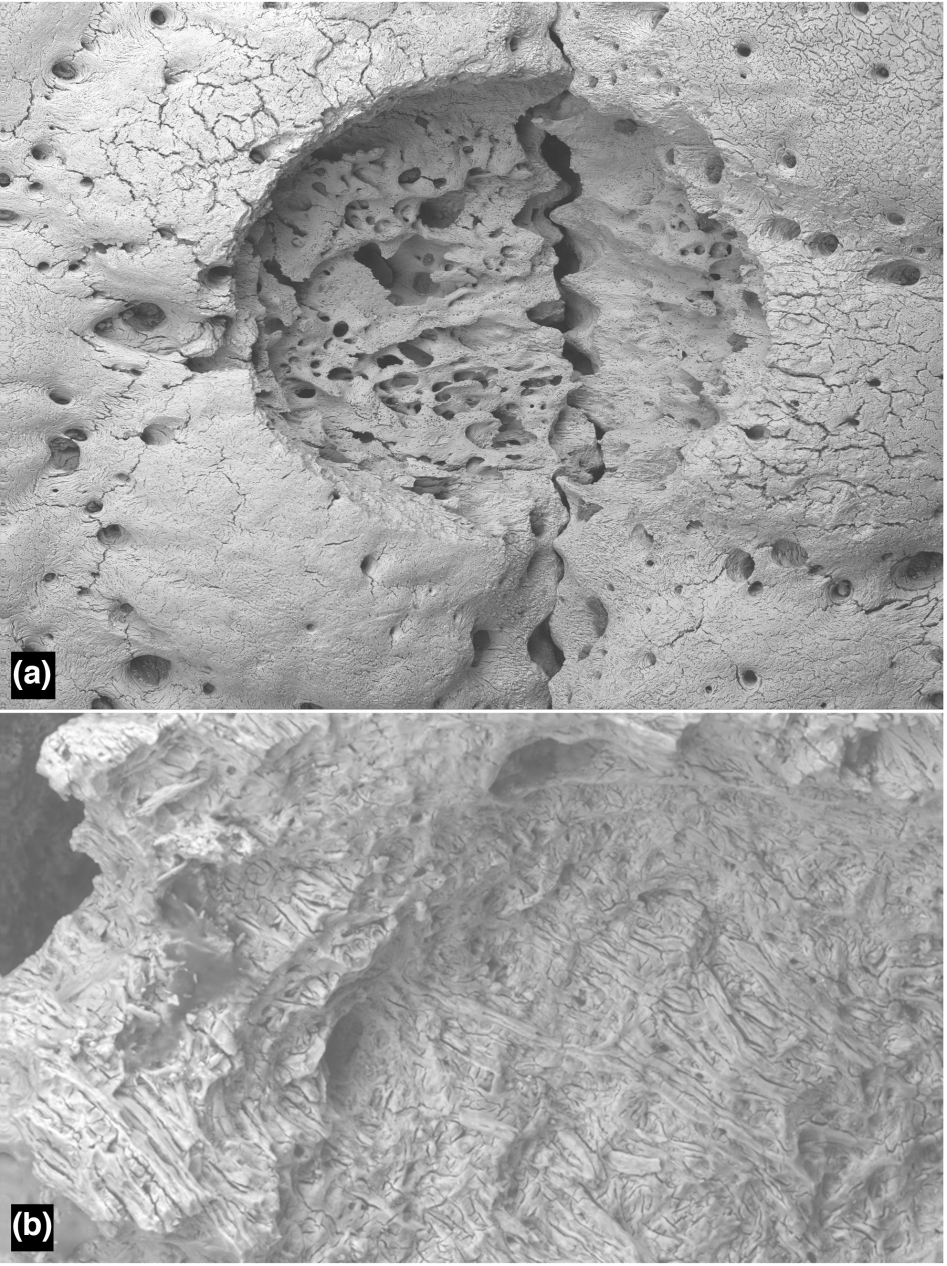
Chaetophractus villosus scanning electron microscopy. (a) Lesion at a suture. Field height 3.6 mm. (for 3D see Figure S6a). (b) Resorption of Sharpey fibre bone in the advancing edge of a lesion. Field height = 380 μm. (3D in Figure S6b).

**FIGURE 2:**
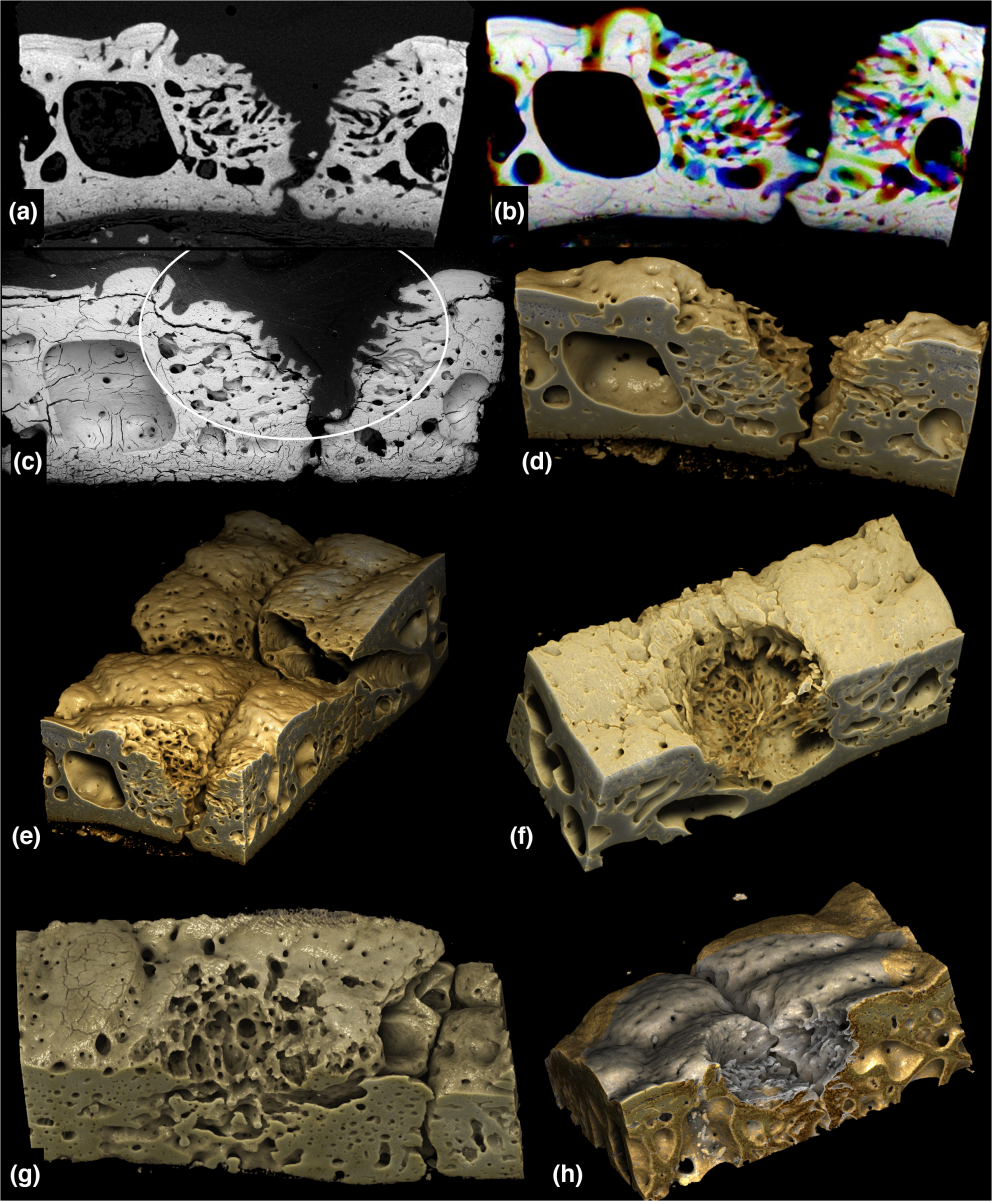
*Chaetophractus villosus*. (a–d) A lesion which has undergone substantial repair. (a) Single 10 μm XMT slice. (b) 18 XMT slices displayed in sequence Red Yellow Green Cyan Blue Magenta. (c) Scanning electron microscopy: ellipse indicates the approximate boundary of the lesion. Field height = 2.59 mm. (d) Drishti rendering of XMT. (e–h) Drishti views of other lesions. (e) Lower face shows cut through a lesion at a suture which has undergone substantial infilling repair: two hair follicle cavities at centre right. (f) Lesion in centre of an osteoderm with early stages of formation of new trabecular bone in its base. (g) One side of a lesion crossing a suture, with hair follicle cavities entering bone surface from the right at the right. (h) Lesion crossing a suture. Patch had been impregnated with Araldite which shows as silvery colour in this Drishti reconstruction. XMT, X‐ray microtomography.

**FIGURE 3:**
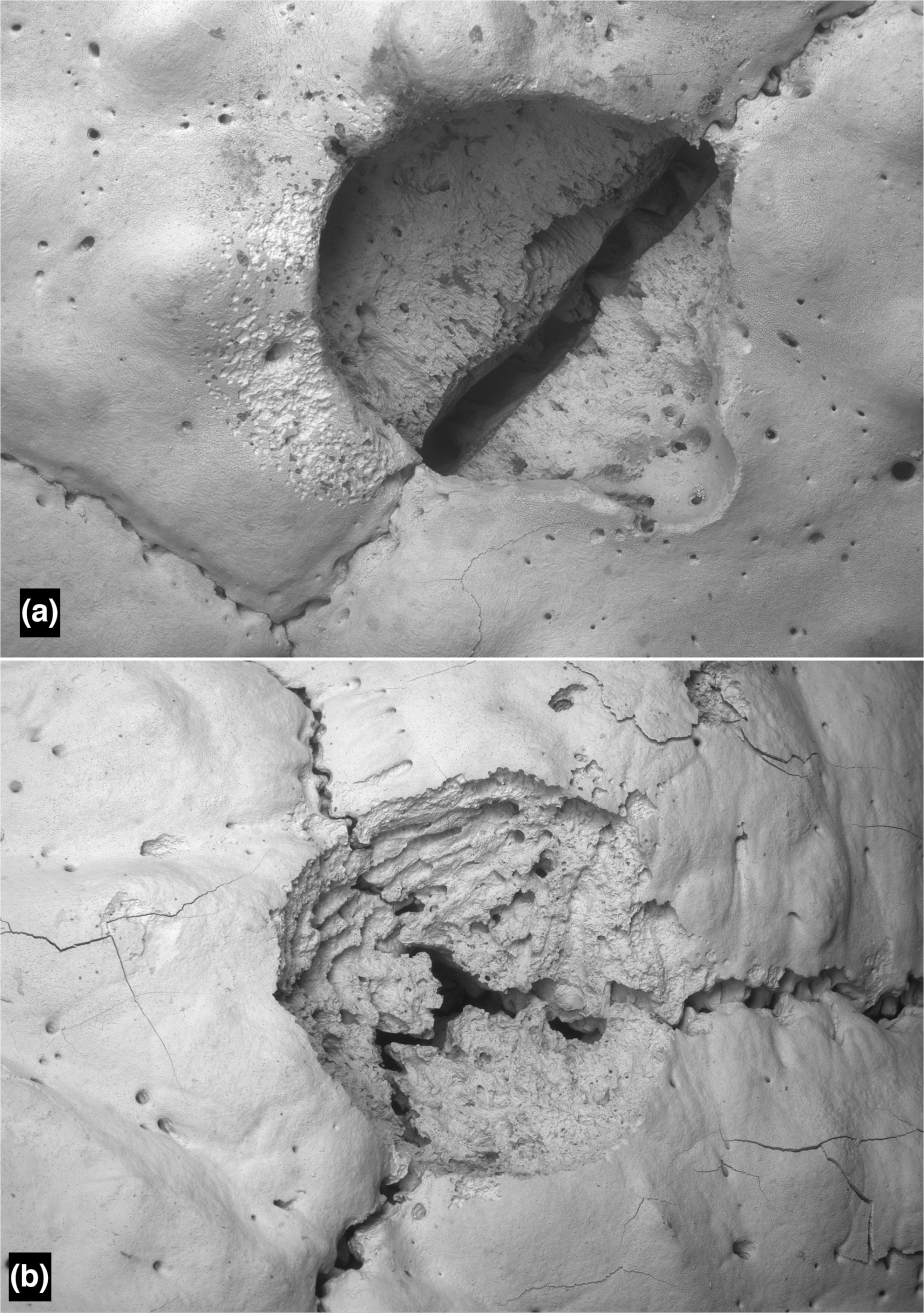
*Tolypeutes matacus*. Scanning electron microscopy (a) lesion at a junction between two osteoderms. Field height = 4.69 mm (for 3D see Figure S7a). (b) Lesion at a triple junction. Field height = 5.32 mm (3D in Figure S7b).

Three‐dimensional BSE–SEM images of the lesions showed resorption pit complexes characteristic of those made by osteoclasts (Boyde, 2019, 2021; Boyde & Jones, 1991; Saftig et al., 1998). Many lesions were centred on the syndesmoses (sutures) between adjacent bones, but some on the centres of bones. Many lesions showed evident signs of repair by deposition of new bone.

The 3D available from XMT showed extra non‐bone space tunnels converging on the lesions, analogous to the micro‐anatomy of overload arthrosis lesions in equine fetlock joints which we have studied extensively (unpublished).

In the hairy armadillo, *C. villosus*, there are numbers of cavities in the distal, backwards‐facing edges of the osteoderms, which accommodate the hair follicles.

The complicating feature of these piliferous follicle foraminae is not present in *D. novemcinctus* or in *T. matacus* which have no hairs emanating from the carapace.

**FIGURE 4:**
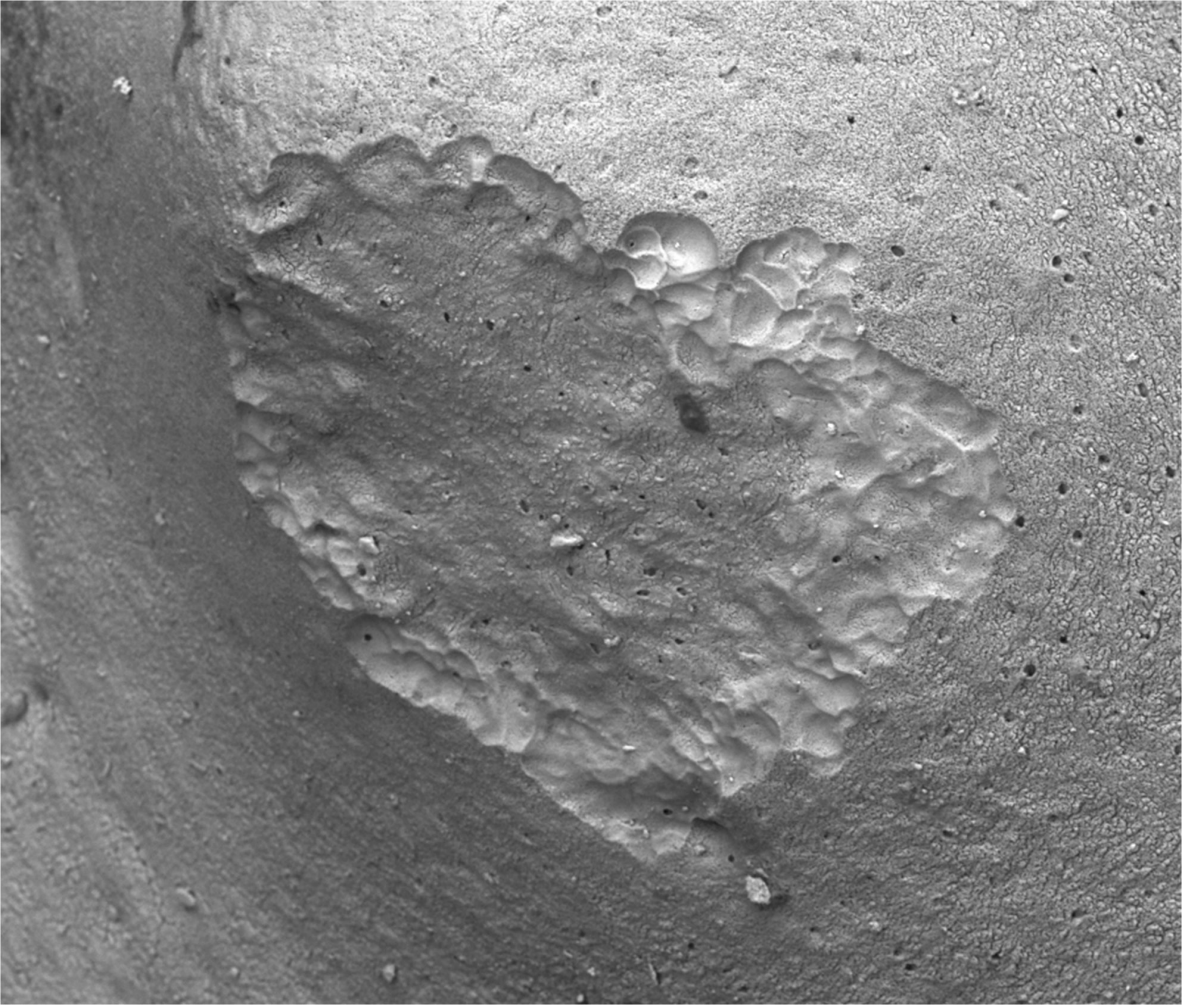
*Tolypeutes matacus*, external surface of an osteoderm with a 0.5 mm wide resorption patch which is infilling with new bone. This is a normal finding, unrelated to the flea‐induced lesions. Scanning Electron Microscopy. Field height = 733 μm.

Small osteoclastic resorption patches, which had been repaired by new bone deposition (resorption–formation coupling), were sometimes encountered on external surfaces of osteoderms in all three species studied, but this we would expect to find when searching on almost any bone in any mammal (Figure 4).

Key features of the surface structure of normal osteoderms in *D. novemcinctus*—which does not have flea bite lesions—are shown in section the in two‐dimensional compositional contrast BSE–SEM in Figure S11a and in 3D topographical contrast BSE–SEM in Figure S11b. Figure S11c shows a ground section viewed with multirotation polarised light microscopy.

## 4 DISCUSSION

### 4.1 Fleas in general

Fleas, Order Siphonaptera, are flightless insects, parasitic on mammals and birds, in which the adults live by sucking blood. Eggs are shed by the gravid female whilst on the host. Larvae may remain on the host or live on or in the ground and utilise organic debris as food. After the last instar, the larva pupates. The quiescent pupa may survive for a long period before the final metamorphosis to the imago, or adult form. If this occurred away from the host, the adult has to jump onto the new host.

### 4.2 The sand flea *T. penetrans* and Tungiasis

The true sand flea *T. penetrans* (jigger, chigoe and many other synonyms) is parasitic on many mammalian species, and many tens of millions of impoverished humans are infected in tropical and subtropical latitudes (Eisele et al., 2003; Miller et al., 2019). Tungiasis is thus a serious pathology in man but is also a major problem in domesticated pigs and cattle. *T. penetrans* also affects rats, and its life history and resultant histology has been studied in detail in laboratory rats (Feldmeier et al., 2007). Female fleas which have burrowed their head ends into skin are mated by males in situ. Within the epidermis of the host, they develop a comparatively gigantic swelling of abdominal segments known as the neosome (the process is called neosomy—Audy et al., 1972; Geigy & Herbig, 1949; Gordon, 1941; Linardi & de Avelar, 2014; Pampiglione et al., 2009) and within this grow large numbers, even hundreds, of eggs which are shed, with faeces, from the exposed end of the body. The insect penetrates to the deep layer of the epidermis in man, but through the basement membrane into the dermis in rats (Feldmeier et al., 2007).

### 4.3 Tunga perforans

*Tunga perforans* is a new *Tunga* species, so termed because it perforates the superficial bone of the osteoderms in certain armadillo species (Ezquiaga, 2013; Ezquiaga et al., 2015, 2021). Hammond et al. (2014) show *T. perforans* type lesions in an archaeological context in pygmy armadillo (*Zaedyus pichiy*) osteoderms. Evidence from extinct fossil species shows that similar lesions were made a very long time ago (late Miocene—Tomassini et al., 2016; Quaternary—de Lima & Porpino, 2018; Nascimento et al., 2020). Perea et al. (2020) show, in their figure 5a, an example of a lesion at a triple junction very similar to those which we have found in the present study in their study of insect trace fossils in glyptodonts—an extinct subfamily of heavily armoured, large armadillos. There have been no prior studies of this pathology to date, but Moura et al. (2021) provide a cartoon (their diagram Figure 4) which is directly pertinent to the present study. They show a female flea ‘eating into’ bone, being fertilised, and then its neosome expanding to create a cavity in the external surface of an osteoderm—this in a Quaternary armadillo—and without explaining how the cavity is formed. By implication from the figure, we may be led to believe that part of this process may be due to the female flea literally eating the bone.

### 4.4 Osteophagy by insects

There are reports in the archaeological and palaeontological literature indicating that insects—especially termites and beetles but also other genera—may destroy bone to enter and feed inside a dead bone—perhaps to make space for other members of a colony, or to make pupating chambers (Backwell et al., 2012, 2020; Britt et al., 2008; Derry, 1911; Huchet et al., 2011; Paes Neto et al., 2016; Pirrone et al., 2014; Xing et al., 2015). Britt et al. (2008) provided convincing good‐resolution microscopic (SEM) studies of surface features of bone surfaces ‘gnawed’ by insects. Accepting that it does occur, then chitinous insect jaws must be able to excavate calcified bony tissues as well as any surrounding non‐calcified connective tissues (tendon, ligament, fibrous periosteum). That is not to say that some of the features supposed to be due to insect boring and grooving activity could be quite normal bone structural characters and some may have been the result of post mortem fungal boring activity (Bell, 1995; Hackett, 1981; Hagelberg et al., 1991). Nevertheless, some structures tentatively ascribed to post mortem insect jaw activity at internal and external surfaces of bones of the endoskeleton in dinosaurs may be like the *T. perforans* induced lesions in armadillo osteoderms. (Pirrone et al., 2014). Thus, the point needs to be considered, could the female *T. perforans* chew bone to make space for the expansion its incipient neosome?

### 4.5 Possible mechanisms for the lesion formation

It would seem to us unlikely that the gravid—or gravid‐to‐be—female flea, having penetrated the epidermis, could eat its way through any bony tissue to create a hole of the size and shape of the later‐to‐be‐formed neosome, albeit that such bone would have been alive, not dehydrated and not hardened by drying.

Fleas feed on blood, not bone. In so doing, they have to prevent blood clotting and produce powerful anticoagulant(s) (Lu et al., 2021, 2022). Anticoagulants such as heparin cause osteoporosis (Nelson‐Piercy, 1997, 1998; Signorelli et al., 2019). Heparin directly augments osteoclastic resorptive activity (Fuller et al., 1991).

Inflammation has a strong association with bone resorption (Terkawi et al., 2022; Yamaguchi & Fukasawa, 2021). Could other factors injected by the flea stimulate inflammation and hence resorption? Inflammation can also be caused by a superadded bacterial infection as in periodontal disease or by pressure due to swelling such as a tumour. With or without inflammation, the work of bone tissue removal is done by osteoclasts. They leave the hallmark imprint of resorption pits and pit complexes which can be recognised in 3D images of bone surfaces (Boyde, 2019; Boyde & Jones, 1991; Saftig et al., 1998). Using 3D SEM, we identify this characteristic bone surface in the flea‐induced lesions in the armadillo osteoderms. SEM of surfaces—rather than any method which involves the disruption of the sample by sectioning—is a very efficient system for searching for and mapping areas where resorption has occurred.

Bone resorption in vivo can be initiated by pressure in the vicinity of a bone surface, as in clinically induced orthodontic tooth movements in man and in normal eruptive and mesial drift movements of teeth in alveolar bone in man and other mammals. We can compare the expanding neosome to a balloon being inflated below the epidermis and in nearby relationship to a periosteum. Judging by the histology shown for *T. penetrans* in situ in rat and in man, the insect's body, at the neosome, is an implant which is covered in a thin stratified squamous epithelium (Feldmeier et al., 2007).

From the present observations, we conclude that the flea which causes the holes in the armadillo osteodermal armour does not itself attack bone tissue directly. From the morphological data, we can see that bone removal must have been conducted by osteoclasts, a process which we and others have studied extensively in many mammalian species, in vivo, ex vivo, in vitro and in silico (Boyde, 2019, 2021; Boyde & Jones, 1991; Saftig et al., 1998). We conclude that the *T. perforans* neosome, or its presence, creates a local host inflammatory response which causes the recruitment of osteoclasts and the resultant bone resorption, creating the space in which it can grow. The histology, biology and molecular biology of the sequence of events will be a challenge for future investigations using fresher or fresh living tissue samples.

The 3D morphology of the bony lesions as revealed by both SEM and XMT show remodelling—reworking—of the surrounding bone structure with the development of large blood vessel canal spaces—a process itself requiring substantial bone resorption—which focus onto the lesion. In this respect, the lesions resemble the re‐organisation of bony tissues seen in naturally occurring osteoarthritis in both man and horse (Boyde, 2021). Such remodelling again indicates that forces other than the insect's own mouth parts are involved in lesion formation, which, we conclude, is work done by and the response of the host's own bone‐related cells. Further, owing to the superficial location of these lesions, which gives a natural observational window into bone (Boyde et al., 1995), we speculate whether they might constitute a useful natural, ‘model’ for future in vivo observation of events occurring during bone modelling.

Another future challenge is to understand the effect of these lesions on armadillos' behaviour and health, taking into account the other lesions of *T. penetrans*, and possibly another species of *Tunga* that penetrate into soft tissues, causing erythema, oedema and pain in the host and in extreme cases producing considerable necrosis and bacterial superinfection (Feldmeier et al., 2004, 2007).

## AUTHOR CONTRIBUTIONS

The study was conceived and study material was obtained by Alan Boyde, Agustin Manuel Abba and María Cecilia Ezquiaga. Alan Boyde did the sample preparation and microscopy and drafted the manuscript. XMT was conducted by David Mills. All the authors contributed to review and approval of the manuscript.

## CONFLICT OF INTEREST

The authors have no conflict of interest.

## Supporting information


Data S1.

## Data Availability

The data that support the findings of this study are available from the corresponding author upon reasonable request.
