# Supplementary material for: Fleas and lesions in armadillo osteoderms
Source: J Anat. 2023 Mar 2;242(6):1029–36. doi: 10.1111/joa.13842 (PMC10184550; doi:10.1111/joa.13842)
Supplement: Supplementary file 1 — Data S1. [file JOA-242-1029-s001.zip › joa13842-sup-0008-Suppl Figure Legends.docx]

***Supplementary Figure Legends***

**Supplementary Figure 5.** Chaetophractus villosus, greater hairy armadillo. **A**. Photo taken with light from top right corner, giving strong shadow contrast. A neosome body (left of centre) has been dislodged from the defect seen to the right. Note the hairs growing out from the distal (towards bottom of the image) edges of the osteoderms. Field width 21.5 mm. **B**. Photo of a large sample mounted for BSE-SEM imaging. Scale bar length 40 mm in top of image field. Red circles around lesions. **C**. Montage of 209 SEM images of same. **D**. Photo after Araldite epoxy resin glue was added prior to sawing smaller specimens for XMT.

**Supplementary Figure 6.** Chaetophractus villosus, 3D BSE-SEM. Pairs of images recorded at 6º tilt angle difference are presented as anaglyph 3D images for viewing with red and cyan filter spectacles, red for the left eye. This method of 3D viewing works well with a computer screen irrespective of colour blindness. **A**. Lesion at a suture. Field width 3.6 mm. Same field as main text Fig 1A, rotated 90º. **B**. Resorption of Sharpey fibre bone. Field width = 360 μm. Most of field shown in Fig 1B, rotated 90º.

**Supplementary Figure 7.** Tolypeutes matacus, southern three-banded armadillo. 3D BSE-SEM. Anaglyph stereos. **A**. Lesion at a junction between two osteoderms. Field width = 5 mm. Same field as main text Fig 3A, but rotated 90º. **B**. Lesion at a triple junction. Field width = 5.7 mm. Same field as Fig 3B, rotated 90º.

**Supplementary Figure 8.** Chaetophractus villosus, 3D BSE-SEM of resin casts of spaces in bone. **A**. Araldite cast of a lesion. Field height = 3.11 mm. **B**. PMMA cast of medullary spaces in normal osteoderm. Colour is coding for direction of BSE collection. Field height = 1.5 mm.

**Supplementary Figure 9.** Tolypeutes matacus, matched Drishti XMT (**A**,**C**,**E**) and SEM (**B**,**D**,**F**) views of a deep, unrepaired lesion at a triple junction, with extensive openings into the medullary spaces of the osteoderms. Field heights of SEM images B = 4.469 mm; D = 5.044 mm; F = 5.556 mm.

**Supplementary Figure 10.** Tolypeutes matacus, same lesion as shown in Suppl Figure 9. 3D BSE-SEM anaglyph stereo, field width = 4.9 mm.

**Supplementary Figure 11**. Dasypus novemcinctus osteoderms. NB Flea induced lesions have not been reported in this species. **A**. Polished PMMA block face prepared parallel to the body surface at a triple junction, coated with carbon and imaged with 20 kV BSE-SEM, showing compositional contrast: whiter means more densely mineralised. NB uncalcified tissue is not seen in this imaging mode. Field width = 849 μm. **B**. Surface facing a suture in an anorganic preparation. 3D topographic contrast. Mineralised portions of the most superficial Sharpey fibres define the boundaries of half-formed osteocyte lacunae. Field height = 179 μm. **C**. Ground section, viewed with multi-rotation PLM: maximum values from 6 images at 15º rotations of crossed polars (see Kirby et al., 2020). Field width 1220 μm.
