# Supplementary figures and images for: Fleas and lesions in armadillo osteoderms
Source: J Anat. 2023 Mar 2;242(6):1029–36. doi: 10.1111/joa.13842 (PMC10184550; doi:10.1111/joa.13842)

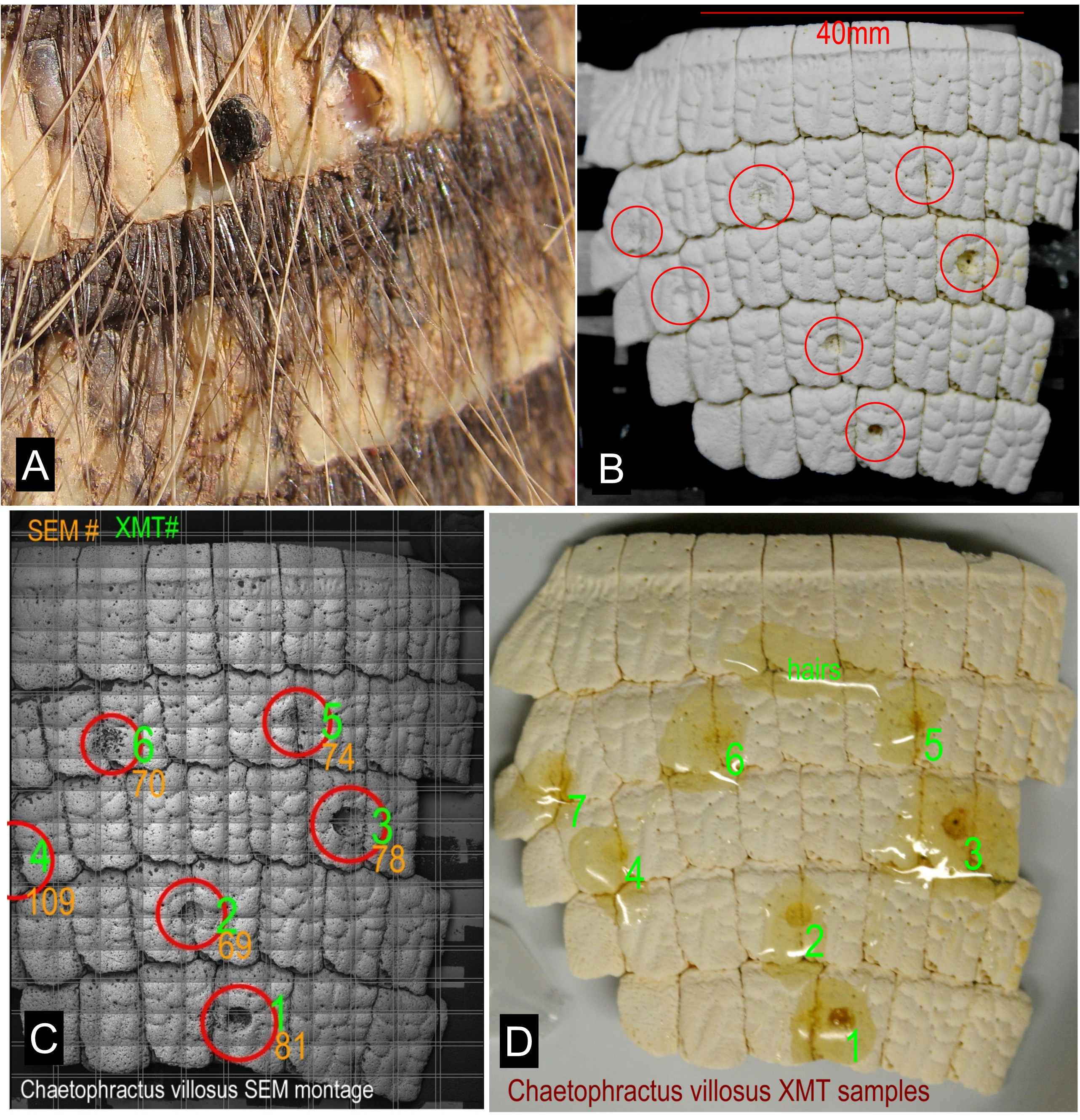

Supplement: Supplementary file 1 — Data S1. [file JOA-242-1029-s001.zip › joa13842-sup-0001-Suppl Figure 5.TIF]

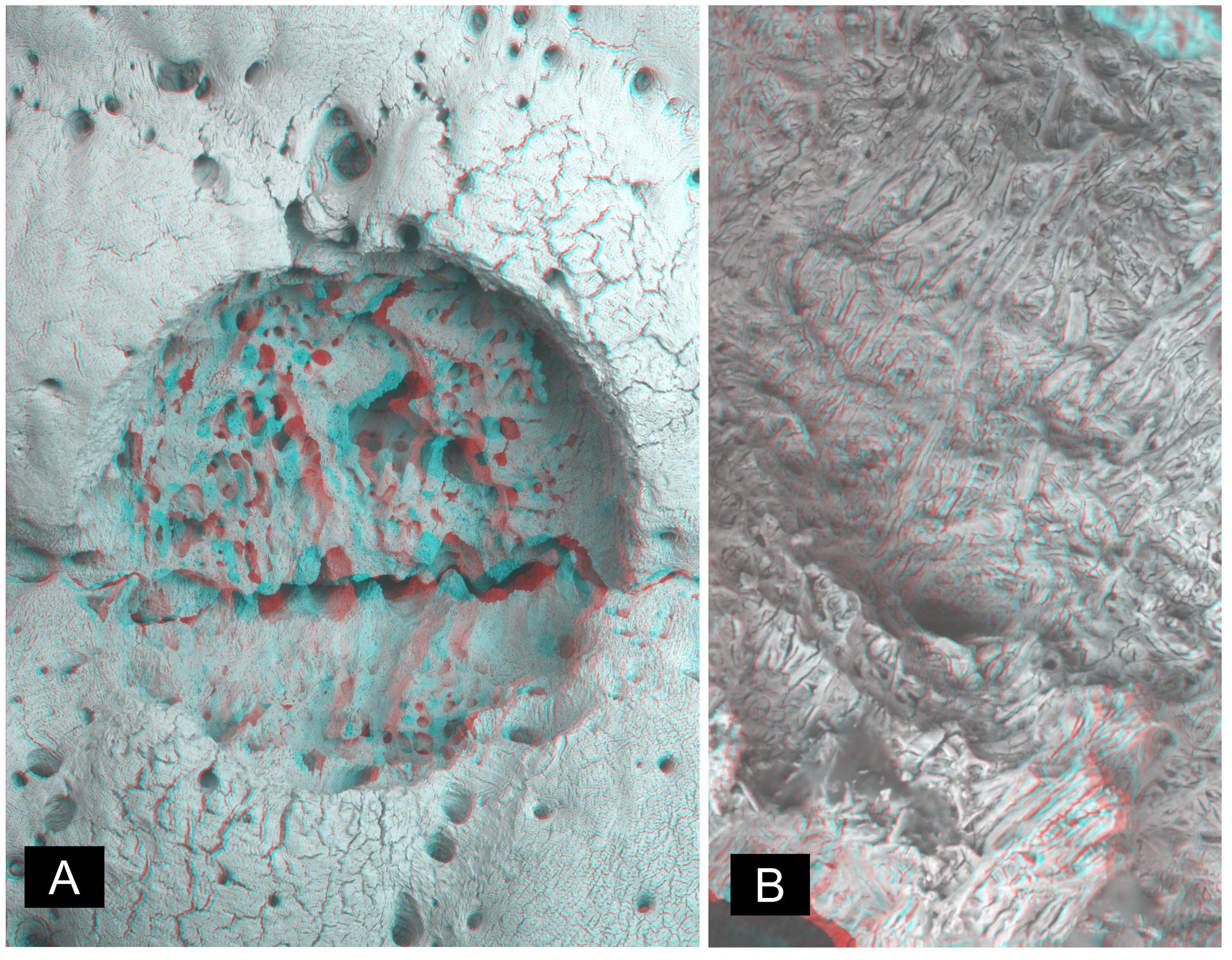

Supplement: Supplementary file 1 — Data S1. [file JOA-242-1029-s001.zip › joa13842-sup-0002-Suppl Figure 6.TIF]

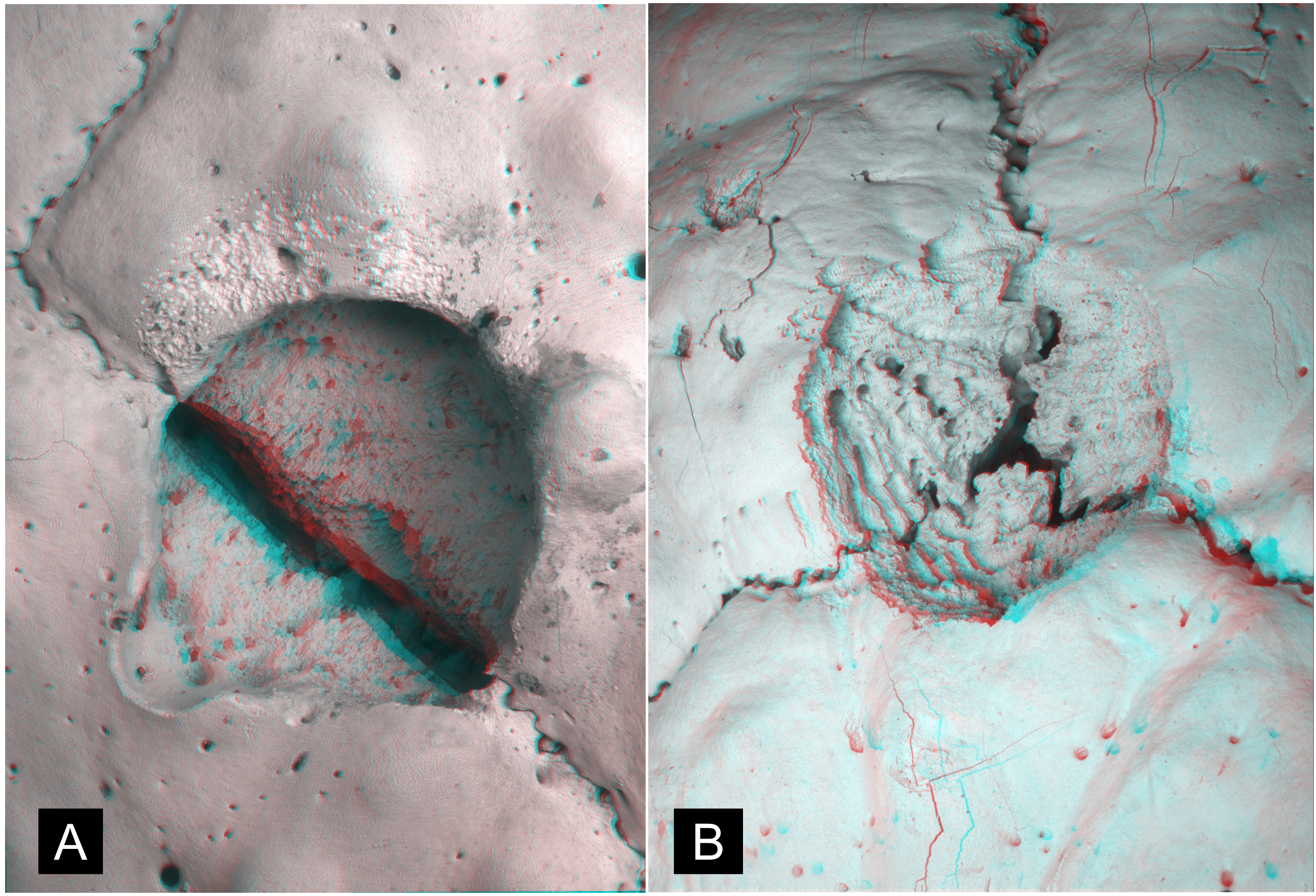

Supplement: Supplementary file 1 — Data S1. [file JOA-242-1029-s001.zip › joa13842-sup-0003-Suppl Figure 7.TIF]

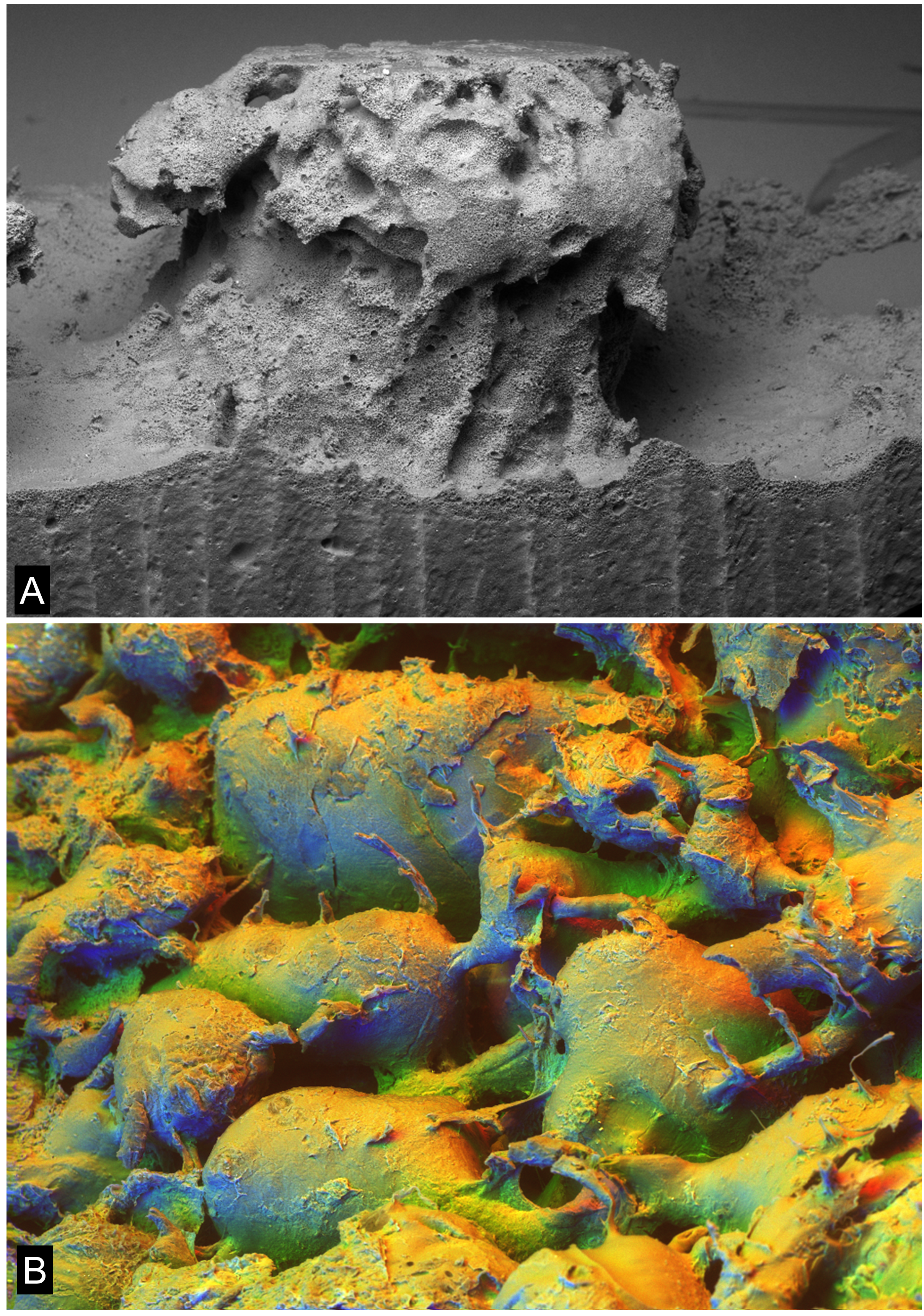

Supplement: Supplementary file 1 — Data S1. [file JOA-242-1029-s001.zip › joa13842-sup-0004-Suppl Figure 8.TIF]

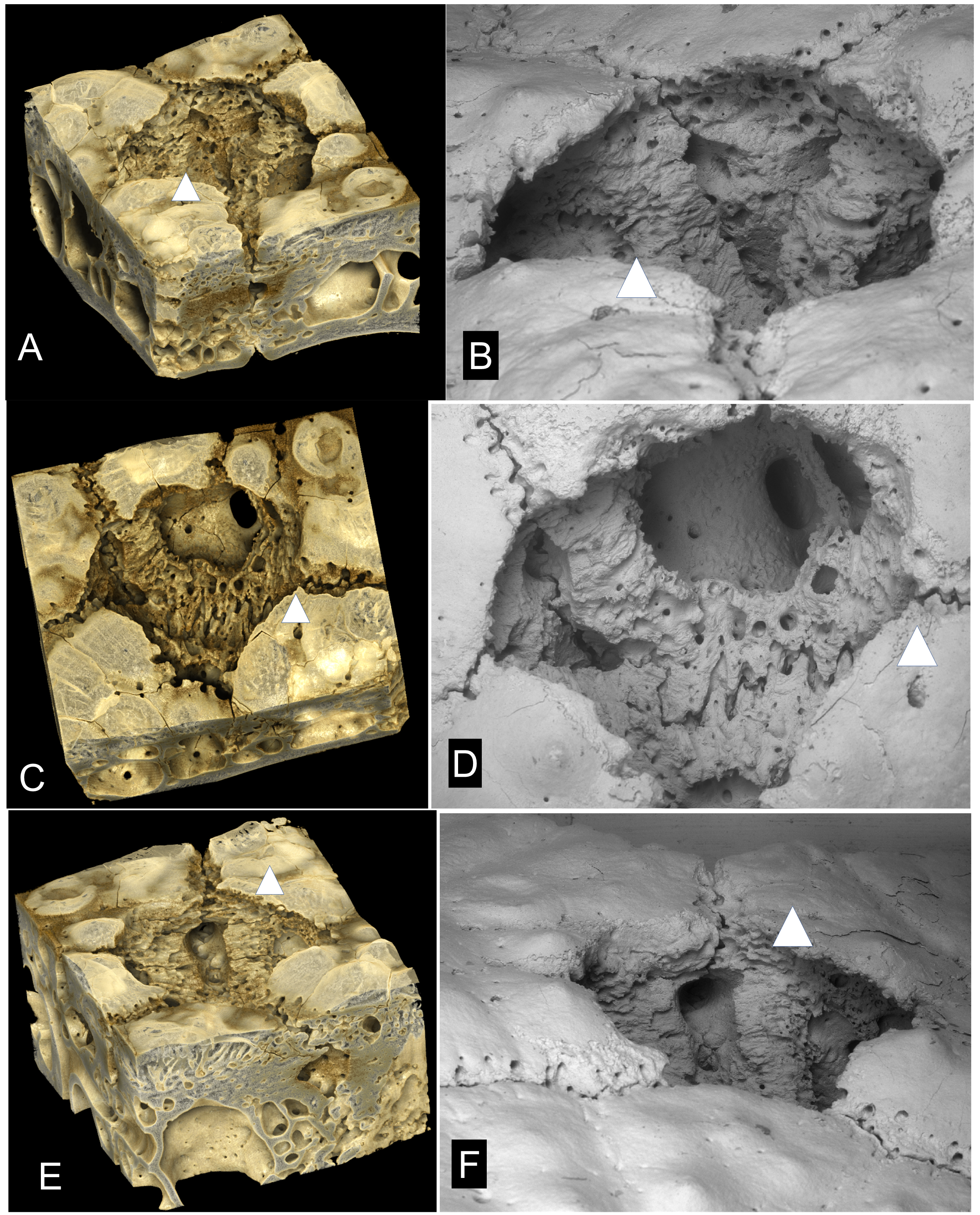

Supplement: Supplementary file 1 — Data S1. [file JOA-242-1029-s001.zip › joa13842-sup-0005-Suppl Figure 9.TIF]

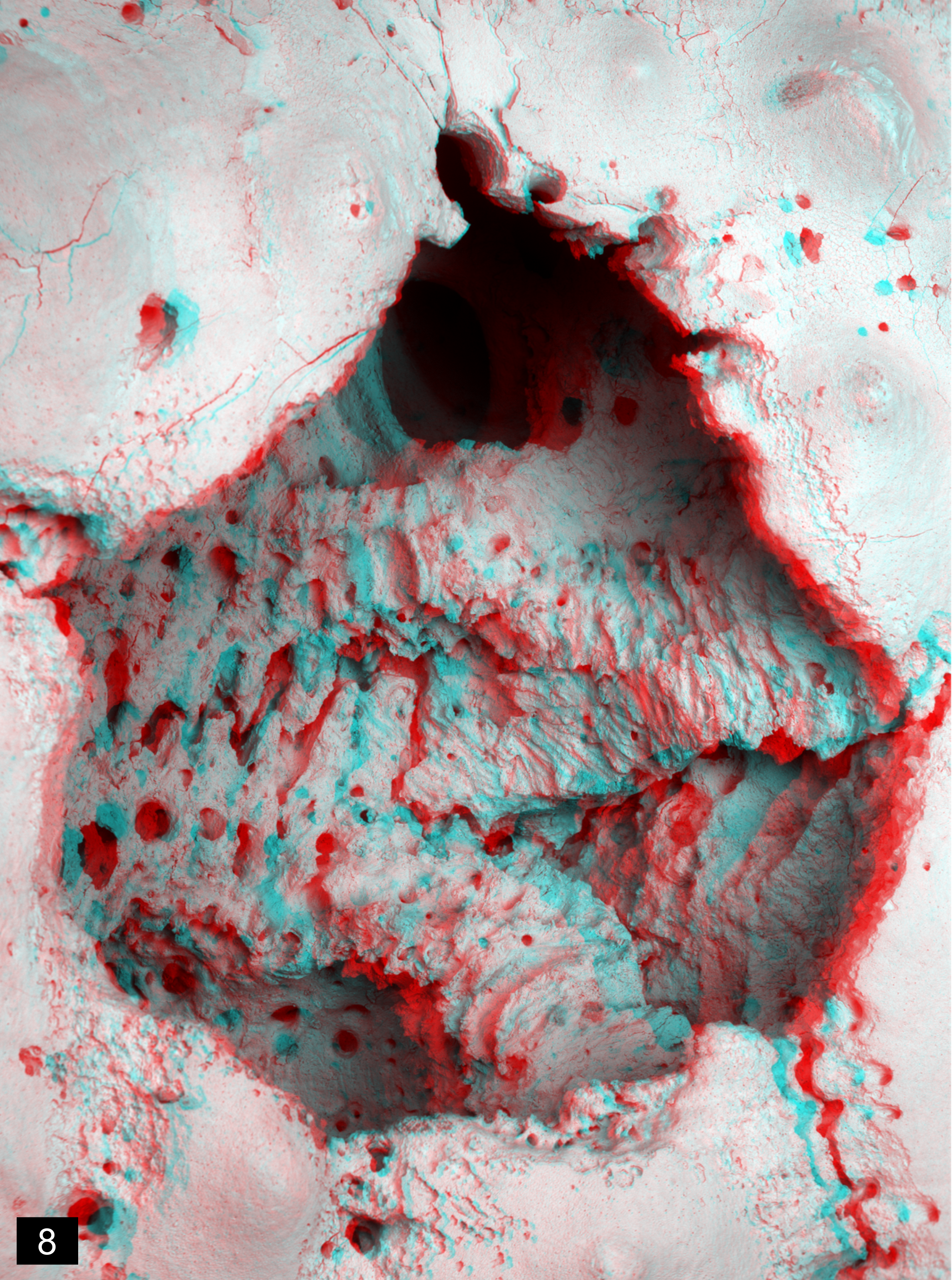

Supplement: Supplementary file 1 — Data S1. [file JOA-242-1029-s001.zip › joa13842-sup-0006-Suppl Figure 10.TIF]

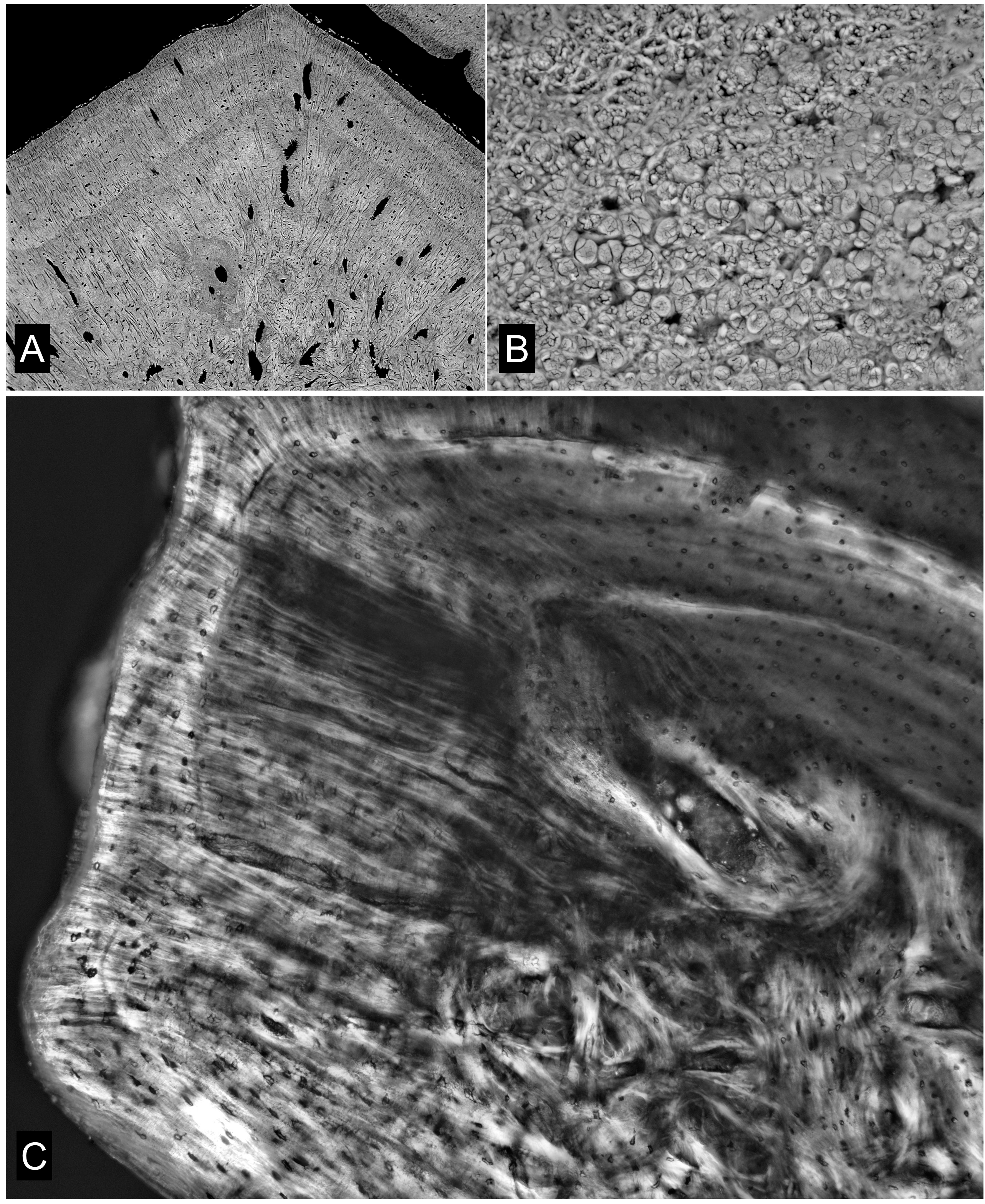

Supplement: Supplementary file 1 — Data S1. [file JOA-242-1029-s001.zip › joa13842-sup-0007-Suppl Figure 11.TIF]
